# An aggressive aneurysmal bone cyst of the proximal humerus and related complications in a pediatric patient

**DOI:** 10.1007/s11751-012-0132-9

**Published:** 2012-03-20

**Authors:** Melih Güven, Murat Demirel, Turhan Özler, Ibrahim Cumhur Başsorgun, Serdar Ipek, Sadullah Kara

**Affiliations:** 1 FEBOT, Department of Orthopaedics and Traumatology, Faculty of Medicine, Yeditepe University, Istanbul, Turkey; 2 Department of Orthopaedics and Traumatology, Ankara Bayındır Hospital, Ankara, Turkey; 3 Department of Pathology, Faculty of Medicine, Akdeniz University, Antalya, Turkey; 4 Department of Orthopaedics and Traumatology, Faculty of Medicine, Abant Izzet Baysal University, Bolu, Turkey

**Keywords:** Aneurysmal bone cyst, Nonvascularized fibular graft, Nonunion, Humerus, Complication

## Abstract

Clinical behavior of aneurysmal bone cyst (ABC) in younger patients can be more aggressive than that in older children and adults. Angular deformity and shortening can occur due to growth plate destruction or tumor resection. A 11-year-old boy who had been operated twice in another center for an ABC located in the left proximal humerus presented to the author’s institution with complaints of pain, deformity and shortening of the left arm. Plain radiographs revealed left proximal humerus nonunion with a large defect. Reconstruction with nonvascularized fibular autograft was applied and left upper extremity was immobilized in a velpou bandage. At the third-month follow-up, graft incorporation was observed in the distal part; however, proximal part did not show adequate healing on radiographs. Additional immobilization in a sling for 3 months was advised to the patient and his family. However, they were lost to follow-up and readmitted to the author’s institution at the 12th month postoperatively. Radiographs showed failure of the fibular graft fixation and nonunion of the humerus. Autogenic bone grafts, either vascularized or nonvascularized are the best treatment method for the large defects after tumor curettage or resection. Nonvascularized grafts are technically much easier to use than vascularized grafts and provide excellent structural bone support at the recipient side. However, they may take several months to be fully incorporated. In addition, good therapeutic outcomes require patience and collaboration with the patient and parents. Most importantly, the patient should be monitored closely.

## Introduction

Aneurysmal bone cyst (ABC) is a rare benign osteolytic bone tumor that contains blood-filled cavernous spaces separated by septae containing osteoid tissue and osteoclast giant cells [1, 2]. Patients younger than 20 years are affected most frequently. Although ABC can be seen in the whole skeleton, generally involved location is the metaphysis of the long bones.

Several treatment modalities have been described for ABC such as sole curettage, curettage with cementation or bone grafting, fibrosing agents (Ethiblock or phenol) or bone marrow injections, arterial embolization, adjuvant cryotherapy or radiotherapy, demineralized bone matrix applications and segmental or en bloc resections [1]. Small lesions that exhibit minimal destruction or expansion of cortical bone can be treated with intralesional procedures with or without bone grafting. However, large lesions that cause large areas of destruction or major expansion of cortical bone should be treated more aggresively with using segmental or en bloc resection techniques and reconstruction with structural allo- or autografts. Recurrence rates after different intralesional procedures may rise up to 50–71 % in children younger than 10 years and 75 % in children younger than 5 years [3–5]. On the other hand, resection, which is the primary treatment of expandable lesions, has the lowest proven association with recurrence and is as low as 0 % [6–8]. However, resection can be problematic, especially for the lesions located in functionally important structures such as the proximal humerus.

There are limited number of clinical reports about the analyses of pediatric shoulder girdle ABC in the literature. According to these studies, the clinical behavior of ABC in younger patients can sometimes be more aggressive than that in older children [1, 9, 10]. In the proximal humerus, it is important to determine whether the growth plate is affected or not. Angular deformity and/or shortening can occur due to growth plate destruction or tumor resection. Therefore, preservation of normal growth and shoulder function should be the aim of the treatment, but it cannot always be possible [1].

We presented a pediatric patient who was complicated with nonunion, deformity and shortening after multiple surgeries for an aggressive type of ABC located in the proximal humerus.

## Case report

**Fig. 1:**
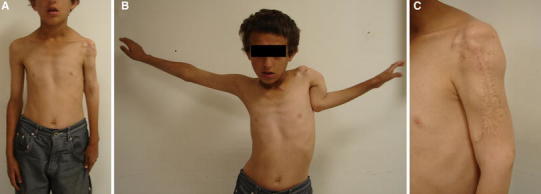
Clinical photographs of the patient show shortening of the *left arm* (**a**), restriction of the *left shoulder* motion (**b**) and old scar tissues on the *left arm* (**c**)

**Fig. 2:**
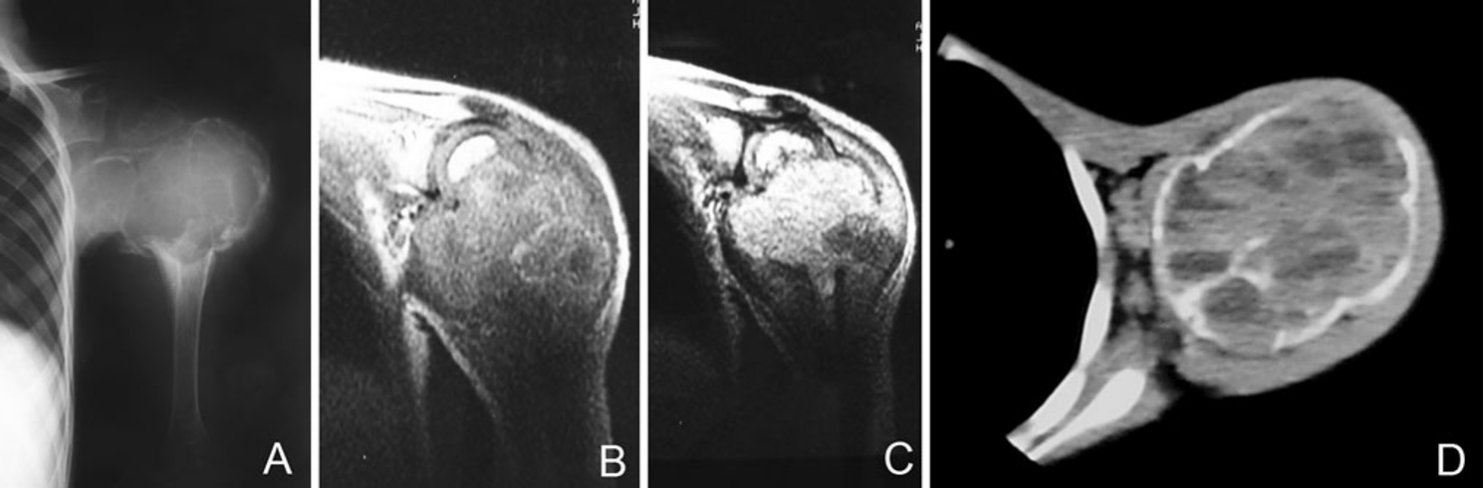
Anteroposterior radiograph (**a**) and coronal MRI scans (**b**, **c**) of the *left shoulder* and *arm* show the blowout appearance of the left proximal humerus. A multiloculated cystic and expansile lesion involving the proximal humeral metaphysis adjacent to the growth plate can be identified. Axial MRI scan (**d**) indicates a fluid–fluid levels in the cystic lesion

**Fig. 3:**
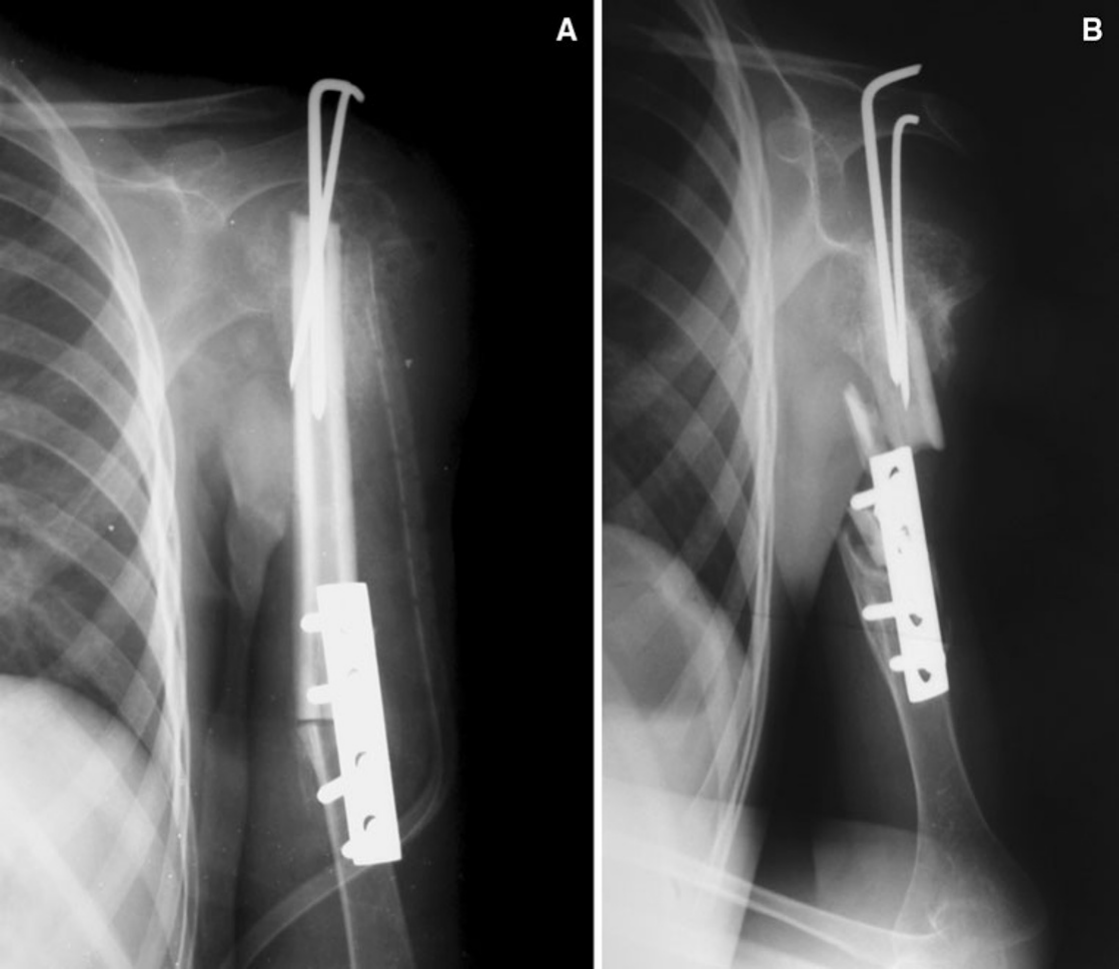
Plain radiograph after initial operation (**a**) indicates en bloc resection of ABC and reconstruction with cortical strut allograft. One year after the index operation, anteroposterior radiograph (**b**) shows failure of fixation with fracture of strut allograft and nonunion of the humerus

A 11-year-old boy presented to the outpatient clinic with complaints of pain, deformity and shortening of the left arm (Fig. 1). Physical examination revealed pathologic motion at the proximal part of the left arm and 8 cm shortening of the left upper extremity compared to the opposite side. According to his past medical history, he had admitted to another institution 4 years before due to pain, swelling and deformity on his left shoulder and upper arm. An expansile, lobulated lytic lesion in the proximal metaphysis of the humerus had been detected on radiographic evaluation (Fig. 2). He had been initially treated by en bloc resection and reconstruction with cortical strut allograft in this center (Fig. 3a). The histopathological results of the tissue samples had been found to be consistent with ABC. Serial follow-up radiographs showed failure of the fixation with nonunion of the proximal humerus (Fig. 3b). Second surgical intervention including excision of the graft material and implants had been applied in the same center, 1 year after the index operation. The defect area had been filled with calcium phosphate allografts, and patient’s arm had been immobilized in a sling. However, union of the humerus could not be achieved.

**Fig. 4:**
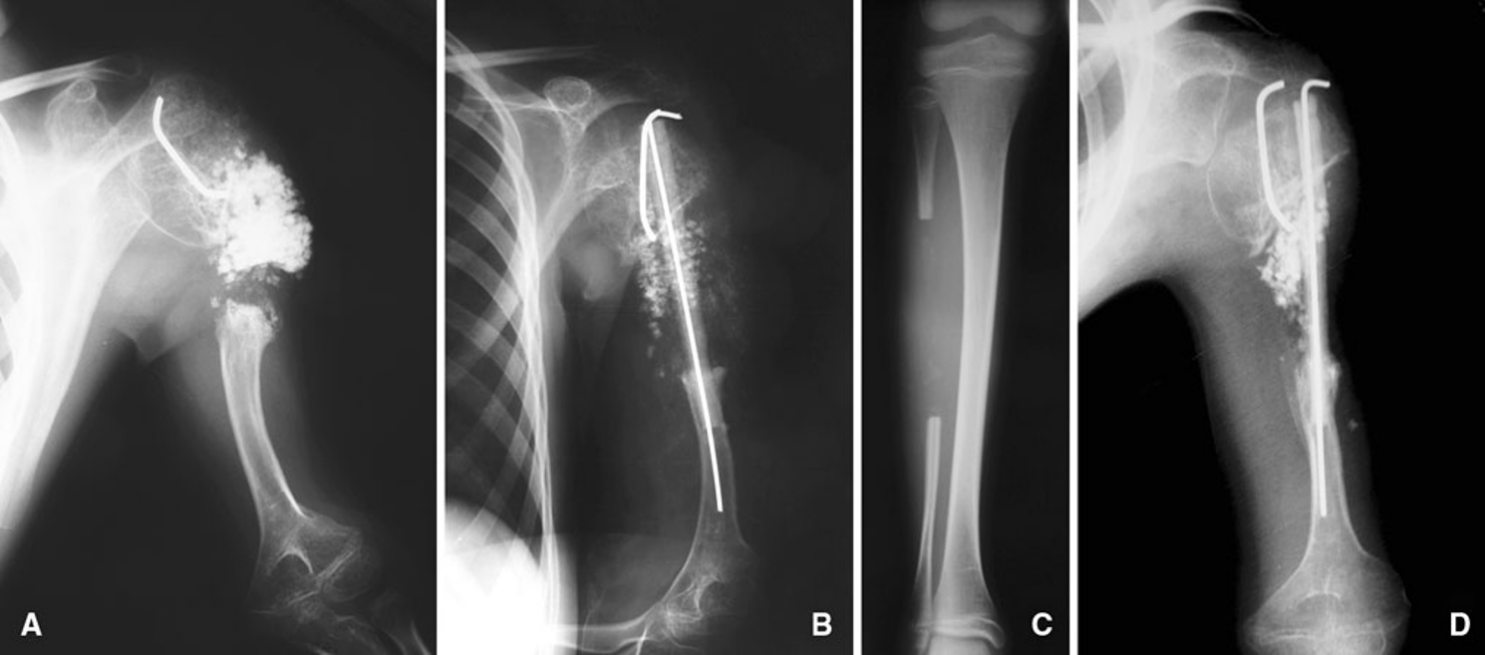
Anteroposterior radiograph (**a**) shows proximal humerus nonunion with a large defect containing allografts. After the operation at author’s institution, plain radiograph (**b**) shows reconstructed defective area with fibular autograft that was secured by one *K* wire. Fibular autograft was obtained from the contralateral extremity (**c**). At the third-month follow-up, plain radiograph shows graft incorporation in the distal part of the fibular graft with inadequate healing of the proximal part (**d**)

Radiographs at the presentation to the author’s institution revealed evidence of proximal humerus nonunion with a large defect (Fig. 4a). His shoulder range of motion was completely restricted with unlimited elbow motion. His neurovascular examination was normal.

Surgery was performed under general anesthesia. The patient was positioned at beach chair position, and deltopectoral exposure over the old scar tissues was used to reach the humerus. Curettage of the dead space, which included the previously inserted allografts, was performed carefully. However, it could not be possible to remove all allograft materials completely due to their adhesions to the surrounding tissues. Then, reconstruction of the defective area with fibular autograft was planned, and the length of the fibular graft was decided. After exposing the contralateral fibular bone, subperiosteal resection of the fibular graft was made under tourniquet control (Fig. 4c). Care was taken to preserve the periosteum in order to provide the opportunity of rebuilding the fibula in the future. The ends of the fibular graft were countered to the shape of the medullary canal of the humerus. The fibula was initially inserted into the distal medullary canal of the humerus and then was placed into the proximal humeral metaphysis. The fixation of the fibular graft was secured by one *K* wire that was inserted intramedullary from proximal to distal (Fig. 4b).

He had an uneventful postoperative course without any postoperative complications. Leg immobilization was not applied. During the postoperative follow-up period, the authors did not observe any nerve injury or palsy in the upper and lower extremities. Immobilization of the left shoulder and arm in a velpou bandage was applied for a period of 12 weeks. At the third-month follow-up, graft incorporation was observed in the distal part of the fibular graft; however, proximal part did not show adequate healing on radiographs (Fig. 4d). Therefore, additional immobilization in a sling for 3 months was advised to the patient and his family. However, the patient was lost to follow-up, and the family did not return to the callings.

**Fig. 5:**
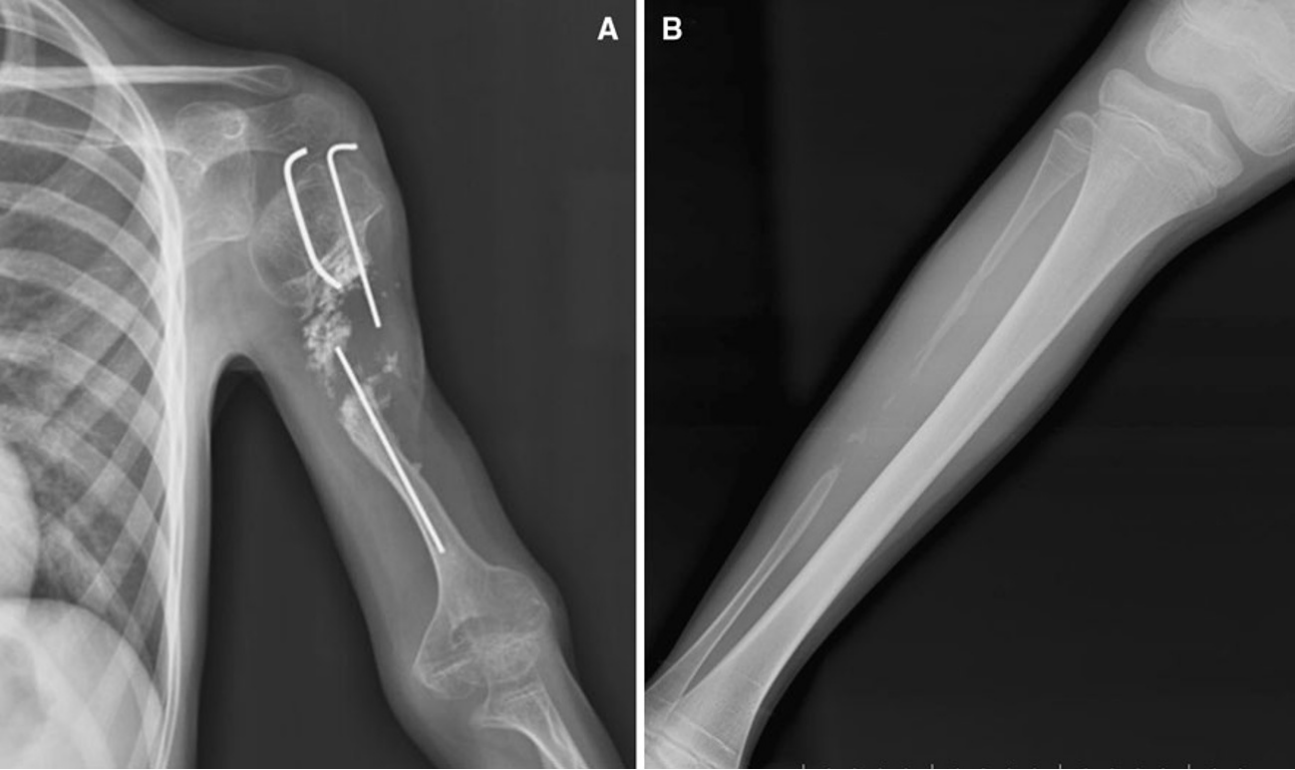
At the end of 12th month follow-up, anteroposterior radiograph (**a**) shows failure of the fibular graft fixation and nonunion of the humerus. Plain radiograph of the *right leg* (**b**) shows new bone formation in the donor side without any angular deformity

The patient readmitted to the author’s institution at the 12th month postoperatively. Physical examination revealed the same findings at the initial presentation and radiographs showed failure of the fibular graft fixation and nonunion of the humerus (Fig. 5). His family stated that they did not comply with author’s suggestions, and the patient started to move his arm without using the sling after 3rd month postoperatively. Second surgical procedure was offered to the patients and family. However, they declined surgery. Written informed consent was obtained from the patient’s family for the publication of this case report and any accompanying images.

## Discussion

The treatment of ABC should be individualized depending on the location, aggressiveness and extent of the lesion. Although curettage and/or en bloc resection are the treatments of choice for accessible lesions, other treatment modalities including percutaneous intralesional injection, cryotherapy, radiation and embolization have been used for less accessible or recurrent lesions. Chemical cauterization with phenol is recommended for relatively large primary lesion to kill any surface tumor cells of the curetted cavity [11]. Cryotherapy has also been proposed as an adjuvant therapy with surgical treatment to achieve local control [12]. Radiation is used in inaccessible sites where no surgical options are available [11]. Selective arterial embolization is recommended as a procedure for lesions whose location or size makes other treatment modalities difficult or dangerous [2].

Large defects after curettage or resection of aggressive type of ABC, which will produce decreased mechanical support like in the present case, are difficult to treat. Various reconstructive options are available to fill these defects and provide bone integrity. These materials include allogeneic or autogenic bone grafts and many different bony substitutes.

Cortical strut allografts have an important role in the treatment of large benign bone lesions after resection [13]. Although they have the advantage of unlimited supply without additional donor site morbidity, the incorporating process of allografts is slower and probably less complete than that with autografts due to a low-grade immune response or a lack of osteocytes in the graft or both [14, 15]. In the present case, allograft material that was preferred as a first choice for reconstruction should be criticized. However, most importantly, the unstable osteosynthesis used in the initial operation was responsible for the failure of graft material.

An autogenic bone graft using the fibula or rib, either vascularized or nonvascularized, has been suggested as the best treatment method for the large defects [16]. Vascularized grafts do not rely on revascularization, and therefore, they become fully incorporated sooner. This technique avoids necrosis and resorption of the incorporated graft and the new osteogenic process; hence, it does not weaken. It remodels in a similar way to the normal bone and is superior to the nonvascularized bone graft [16, 17]. The initial surgical intervention in the present case might be vascularized fibular grafting after en bloc resection of ABC.

However, vascularized fibular grafting is not without problems. It is a technically demanding and time-consuming procedure with a high rate of thrombosis of the graft vessels and is not available in every center [16, 18, 19]. In addition, vascularized fibular graft requires a well-equipped medical team with special microsurgical techniques and a relatively long operative time, further stressing the patient [18]. In the present case, surrounding soft tissue adhesions and disrupted anatomy of the arm due to the previous operations could complicate vascularized fibular grafting.

Nonvascularized grafts are technically much easier to use than vascularized grafts and provide excellent structural bone support at the recipient side [16]. Successful long-term results of surgical en bloc resection and replacement with nonvascularized, autologous fibular or tibial graft have been reported in the literature for the treatment of large benign humeral lesions [9, 10, 16]. Grezegorzewski et al. [9] reported the long-term results in 20 patients with unilateral benign humeral lesions where steroid injection, curettage and bone grafting or pathological fracture failed to restore bone integrity. Lesions included ABCs, solitary bone cysts and fibrous dysplasia. No recurrence of the pathology, pain, graft fracture or limitation in range of motion were noted. There were 2–6 cm shortening of the humerus in three cases at the time of the last follow-up examination. Humeral shortening was observed only in those patients in whom the cyst was adjacent to the proximal growth plate of the humeral bone.

On the other hand, nonvascularized grafts may take several months to be incorporated during which time they lose much of their strength and are susceptible to fracture and/or nonunion. In the first few weeks immediate postoperatively, the mechanical strength of the graft is reduced. The full mechanical properties return after 6–12 months [16]. Abuhassan and Shannak [20] reported the results of nonvascularized fibular graft for the reconstruction of bone defects after en bloc resection of giant ABC in three patients. They observed insufficient graft incorporation at the distal part of the fibular graft in the humerus case at the 18th month postoperatively. They treated this patient by open reduction and internal fixation with additional bone grafting. They advised rigid fixation of fibular graft onto the normal bone with a supplemental form of internal fixation to prevent graft insufficiency. In the present case, graft incorporation was observed only in the distal part of the fibular graft at the third-month follow-up. Therefore, immobilization period was decided to prolong for additional 3 months. But the patient and his family could not show compliance with prolonged immobilization.

The goal of surgery in aggressive type of ABC should be to cure the lesion, achieve adequate stability and prevent refracture or recurrence. If any allograft material is to be used for the reconstruction of the defect, then it should be fixed very stable as much as possible. In the present case, an autogenic bone either vascularized or nonvascularized would be a better option. In addition, trying to obtain functionally mobile, active joint and extremity is also important. However, our functional expectations were limited due to the previous operations.

Good therapeutic outcomes require patience and collaboration with the patient and parents in such cases. The patient’s family should be informed about the high probability of complications and warned about the possibility of staged surgical procedures. Most importantly, the patient should be monitored closely.
